# The Uses of Emotion Maps in Research and Clinical Practice with Families and Couples: Methodological Innovation and Critical Inquiry

**DOI:** 10.1111/famp.12096

**Published:** 2014-08-05

**Authors:** Jacqui Gabb, Reenee Singh

**Affiliations:** * Dept of Social Policy and Criminology, The Open UniversityMilton Keynes, UK

**Keywords:** Emotion Maps, Family Relationships, Visual Methods, Family Systemic Psychotherapy, Clinical Practice

## Abstract

We explore how “emotion maps” can be productively used in clinical assessment and clinical practice with families and couples. This graphic participatory method was developed in sociological studies to examine everyday family relationships. Emotion maps enable us to effectively “see” the dynamic experience and emotional repertoires of family life. Through the use of a case example, in this article we illustrate how emotion maps can add to the systemic clinicians’ repertoire of visual methods. For clinicians working with families, couples, and young people, the importance of gaining insight into how lives are lived, at home, cannot be understated. Producing emotion maps can encourage critical personal reflection and expedite change in family practice. Hot spots in the household become visualized, facilitating dialogue on prevailing issues and how these events may be perceived differently by different family members. As emotion maps are not reliant on literacy or language skills they can be equally completed by parents and children alike, enabling children's perspective to be heard. Emotion maps can be used as assessment tools, to demonstrate the process of change within families. Furthermore, emotion maps can be extended to use through technology and hence are well suited particularly to working with young people. We end the article with a wider discussion of the place of emotions and emotion maps within systemic psychotherapy.

## Introduction

Emotions have had a somewhat vexed history in the field of systemic psychotherapy. Much of the earlier systemic thinking appears to have marginalized emotions, perhaps as a way of distinguishing systemic psychotherapy from psychoanalytic approaches. At the same time, pioneers in the field like Salvador Minuchin (1975) and Virginia Satir (1972) relied on techniques like enactments and sculpts that were based on working with emotions in the room. More recently, emotions have been seen as relational, embodied, and culturally determined (Bertrando, 2008; Fredman, 2004). In this article, attention is focussed on how to bring to the fore and work with emotions as they are experienced in family relationships, at home. To this end, a new technique, the emotion map method, is introduced. This graphic participatory method was developed by author Jacqui Gabb in academic family studies of everyday family relationships (Gabb, 2008); this article will explore the utility of the method in clinical practice with families and couple relationships.

Emotion maps are designed to elicit information on family processes as experienced through interactions, located in the family home. They are not reliant on language skills and therefore can be completed by parents and children alike, irrespective of age or literacy skills. Indeed, children find the method particularly engaging as their perspective can be equally heard. The production of emotion maps can encourage critical personal reflection and interpersonal dialogue. Identifying strengths and weaknesses in a relationship and/or family empowers the participant/client as it wrests control away from experts. The sharing of individual emotion maps between the couple and among family members encourages critical reflection on how events and emotions may be perceived and experienced differently. Importantly, the method centers analytical attention on feelings and the emotionality of family relationships rather than getting mired in particular events or scenarios as “the problem” in and of themselves. For these reasons, as we demonstrate, emotion maps can make a valuable contribution in the clinical assessment and clinical practice toolkit.

In this article, we introduce extant visual methodologies used in work with children and families, situating the emotion map method within this rich methodological vein. The research context of the emotion map technique is presented to illustrate how the method developed through its deployment in empirical studies of couple and family relationships.^1^ It was through her study as a Researcher on one of these projects that author Reenee Singh was familiarized with the emotion map method. The main body of this article presents reflection on the authors’ collaboration, primarily drawing on a case example from Singh's study as a systemic family psychotherapist to demonstrate the applied use of emotion maps in clinical contexts.

## Visual Methods

Visual methods have been routinely used in child development observations, wherein the researcher/clinician watches parent–child interactions while they complete a drawing activity together. The flexibility of visual methods makes it easier for the researcher to take account of children's diversity (Hill, Laybourn, & Borland, 1996) and differences in age/developmental stages. They encourage children to communicate symbolically (Deacon, 2000) and help to overcome generational competencies which ordinarily divide parents and children (Gabb, 2008). They are thus a valuable part of the methodological toolkit in work with children who are preverbal and with both adults and children whose first language is not English or whose language skills are limited (Clark & Moss, 2001). Systemic psychotherapists routinely deploy nonverbal methods in their work with children and families (Wilson, 2007) and participatory action methods have always been used in family therapy (Chimera, 2013). Perhaps the most common visual method used by family therapists is the genogram (McGoldrick, Gerson, & Petry, 2008) or family map; this has proven highly successful in clinical assessment, couple therapy, clinical supervision, training, and clinical research. Genograms provide an opportunity to engage with children and young people in family therapy sessions and in clinical research. The cultural genogram (Hardy & Laszloffy, 1995) adds another dimension to clinical assessment and enquiry and has proven successful in work with intercultural couples, migrant families, and in clinical supervision. In dialogical systemic practice, Rober (2009) has used relational drawings to probe couple relationships and engage the individuals in couple therapy. Another visual method that has found its way into systemic clinical research is family photographs. Family photographs can shed light on family members’ perceptions of who constitutes family (Rose, 2010), something that is especially useful in work with step-parent families.

Many of the visual and action methods used in family therapy, such as enactments (Minuchin, 1975), semantic polarities, and positioning (Campbell & Groenbaek, 2006), rely on spatial metaphors. Sculpting is another method that has a rich tradition in Systemic psychotherapy (Cade, 1982; Papp, 1982; Satir, 1972). More recently the technique has been extended by Papp, Scheinkman, and Malpas (2013) to help couples overcome impasses in their relationship. While Rober (2009) asks couples to draw images of their relationship and bring them into the session to explore together, Papp and colleagues have a developed the method in ways that create a more visceral immediacy. In their sculpting protocols, they invite the couple to work together to enact metaphors which symbolize their relationship. In this way, they can engage with couples’ embodied emotional experiences of their relationships. In the following sections, we show how the emotion map can make a valuable contribution as part of this rich methodological tapestry in the field of systemic family therapy.

## The Emotion Map Method

Floor plans have been in a diverse range of contexts, ranging from social research of daily family life (Graesch, 2004) to clinical social work assessment and psychotherapy (Jacobson, 1995; White, 1976). Most recently, spurred by developments in emotional geographies, spatial mapping has been used to study love (Morrison, Johnston, & Longhurst, 2013) and household living arrangements (Bridge, 2013). It is also being developed for use in the study of nonfamilial contexts such as homelessness and youth offending. Situating emotions at the methodological and conceptual heart of enquiry is the emotion map method. Pioneered by Gabb (2008) in her research on family relationships, this technique focuses attention onto the materiality, temporality, and emotionality of family lives by generating data on everyday relating practices (Gabb, 2009).

**Figure 1:**
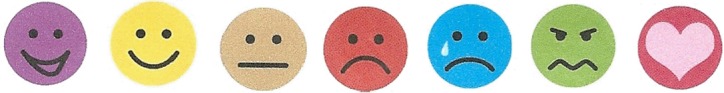
Emoticons

In this context, in research practice, emotion maps are produced over a 1-week period using a set of colored emoticon stickers denoting laughter, happy, indifference, sadness, upset, grumpiness or anger, and love/affection (see Figure 1).

Individuals are asked to place an emoticon sticker on their floor plan as and when interactions occur. Different colors are used for different people, including the participant, their partner, children (if any), family, friends, pets, and so on. The graphic materials produced are then used to facilitate talk on the scenarios and emotions represented. In clinical practice, our use of the method is more immediate with one or more family members being asked to sketch out the floor plan onto which stickers are then placed. In this context, the events and emotions that are depicted are typically cumulative events that happen rather than specific examples as they happen. In the first section of this article, we present one example from research to illustrate the efficacy of the method and also to highlight how the method was developed and tested out in fieldwork.

## Emotion Maps in Research

Emotion maps offer rich analytical rewards in the study of couple and family relationships by building up a picture of the character and quality of everyday experience. However, the breadth of data produced through this method far exceeded initial research expectations. They not only provided information on the patterning of lived relationships, they also facilitated *active* “relationship work.” There is no doubt that for some parents the creation of emotion maps served as a vehicle for them to display consciously crafted parenthood (Gabb, 2013) with recourse to privileged sets of knowledge that foreground child-centered practice (Gabb, 2009) and moral rationality (Duncan & Edwards, 1999). So for example, some parents would talk about the need to manage children's misbehavior through banishment to the “naughty step.” Sometimes seeking to situate themselves in contradistinction to their own experiences as a child, they articulated their parenthood through cultural and theoretical lenses such as “attachment parenting” or “permissive parenting.” In this way, they sought to both validate their role as a “good parent” and gain affirmation of their expertise.

They were used quite differently, however, by parents who had limited access to the cultural capital and social resources required to display such reflexive parenting (Gabb, 2011). Producing an emotion map appeared to afford these parents a chance to think outside the immediate everydayness of family life. For example, Joan, a mother whose experience is shaped by significant social and economic disadvantages, spoke about using the knowledge gained through completing her emotion map to think about how she might proactively redress family problems.

Joan: I found it interesting doing the map, the floor plan, ‘cos I noticed there was patterns in the rooms, especially the kitchen. It seemed like quite an unhappy, grumpy place where I was telling the children off around the fridge, around the cooker and table, the sink. … It starts off quite happy at the table and then we sit down on an evening and by the end of the evening it's just … I realised a lot … I think it was the different areas of the house where the kids must think, ‘This is a grumpy area, she's going to tell us off again now, let's wait for it’, they must just think that, you know, and try and push it as far as they can get….

For this mother, taking part in the research and producing her emotion map was empowering and marked an important family intervention. The experience enabled her to work on changing how her family interacts and thus enhance the parent–child relationship and family life more generally. These “educational” dimensions were wholly unsolicited and initially unexpected; once realized, however, they were followed up and refined in subsequent research (Gabb & Singh, 2014), leading to our shared belief that they held great methodological capacity in applied in clinical contexts. Singh, in her role as a systemic family psychotherapist, then sought to explore their value in these contexts. In the section below, we present a case example that utilizes an emotion map in a family therapy session. In our discussion, we consider its proven potential and further possible uses in clinical assessment and clinical practice.

## Transforming Anger: A Case Example

A colleague referred the Smith family^2^ to Singh because they wanted to “improve couple/family communication.” In the partnership that was established, Singh deployed her expertise as a family systemic psychotherapist to facilitate this process. As part of this process, emotion maps and drawings were used to facilitate personal reflection, family conversation, and “give voice” to the child. The family comprised Beth, a 50-year-old woman who was married to Bill, aged 58. The couple has been married for 13 years; they have a 12-year-old son, Richard, and a 10-year-old daughter, Catherine. Beth has a 21-year-old daughter from a previous marriage who, at the time of referral, was living in a different country. Singh's early hypothesis was that the three children, in different ways, were caught up in their parents’ conflict as they all exhibited symptoms which could be understood as distraction tactics, deflecting attention away from the couple conflict. It was therefore clinically pertinent to offer a combination of couple and family therapy. The latter was specifically requested in the referral. At first, the couple work was framed around working with co-parenting issues, however, in the course of these sessions Singh obtained a contract from the parents to spend some time explicitly focusing on their couple relationship. At the time of writing this article, Singh had completed 10 sessions with the family, over a period of 4 months, working with different sub-systems at different times. Sessions alternated between couple work and family work. The couple had been previously through individual and conjoint therapy but this was their first experience of being seen all together as a family.

**Figure 2:**
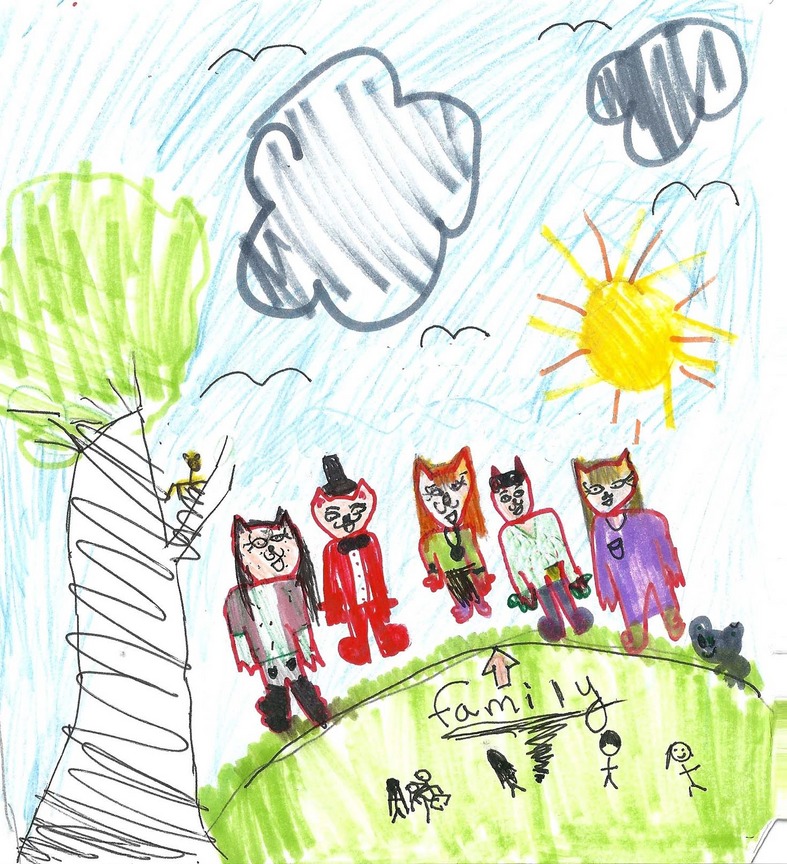
Catherine (Aged 10) Drawing: “My Family”

The presenting problem appeared to be the husband/father's anger and its impact on the rest of his family. Bill had recently taken on a stressful job as the director of an investment bank and was unhappy and somewhat overwhelmed at work. Beth felt that Bill undermined her parenting and was overly harsh with the children, particularly with their son, Richard. In Gabb's research, doorways have been identified as a literal and symbolic frame for family arguments (Gabb, 2008, pp. 138–139). In a similar way, Bill experienced differences in the couple's parenting styles most difficult to negotiate when the family was about to leave the house. He perceived that Beth had “no sense of time” and he frequently lost his temper around the children's lack of organizational skills and apparent inability to make a timely and prompt departure (Figure 2).

During the first session with the Smith family, Singh asked Catherine to draw a picture of her family. She ably expressed herself through drawing and this opened up dialogue among the family group. Catherine's picture depicted the five members of her family and her dog. As the drawing was passed around the family, her brother and parents seemed to fully engage with this representation. This family, therefore, seemed to respond well to visual interventions and so Singh attempted to draw a cultural genogram (Hardy & Laszloffy, 1995) with them. This intervention was less successful. The family did not want to engage with addressing the impact of previous generations in shaping the couple's respective parenting styles.

**Figure 3:**
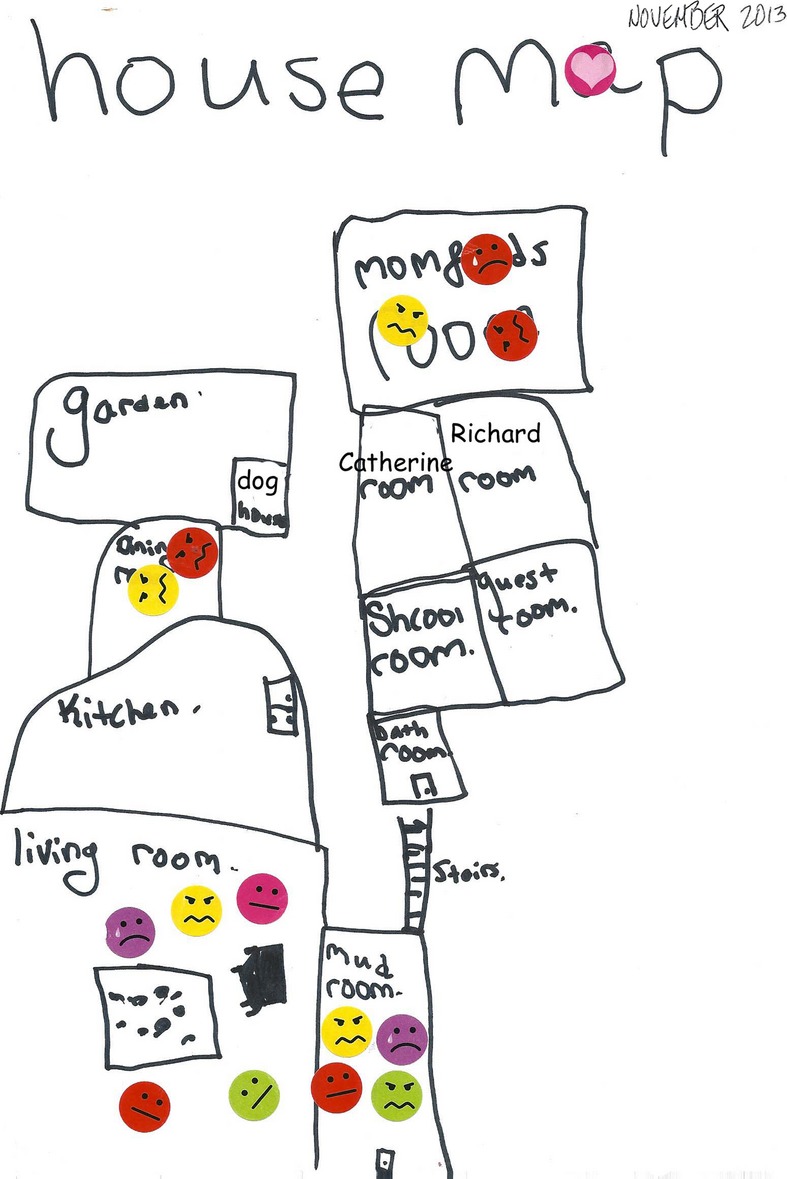
Catherine (Aged 10) Emotion Map 1 Yellow = Dad; red = Mum; Catherine = purple; Richard = green

An impasse was quickly reached, with Beth continuing to feel intimidated and undermined by Bill's anger and Bill feeling unheard and marginalized within the family. Catherine (the youngest family member) took on the role of mediator and peace-keeper and had developed psychosomatic symptoms in response to the anger in the home. Faced with this scenario, Singh introduced the idea of emotion maps to the family. Having experienced the efficacy of emotion maps in a research context, she believed in the capacity of this technique to serve a clinical purpose. Emoticon stickers were brought into the next family session and the technique was fully explained to all family members. Singh made it clear to all family members that participation was optional. It was fine if Catherine did not draw an emotion map; she could instead continue to draw as in previous sessions and thus participate by talking and listening. However, as the group began to talk about a situation where Bill (Dad) had lost his temper, Catherine spontaneously reached for the emoticons and drew (Figure 3).

In this map, the major site of conflict was the “mud room” (hallway), an area where Dad (yellow) and Richard (green) would typically get into screaming rows. Describing events the family agreed that they would be running late for an appointment, a scenario that prompted Bill to lose his temper, focusing his ire on the length of time that it took Richard to put on his shoes. Catherine spoke movingly about how she (depicted in purple) felt sad during these incidents, interactions that repeatedly occurred. Her emotion map shows residual bad tempers lasting long after the event, especially for her Dad, with both parents being depicted as angry in the dining room and in their bedroom. Describing the scenario when her parents are in their bedroom, Catherine remarked perceptively that her Mum (depicted in red) was both angry and sad and this is why she deliberately used these two emoticons.

The family group listened while Catherine described her experience of events and the emotions it summoned. Her account was evidently hard to hear for the parents and her Mum cried all the while. Productively, the emotion map and Catherine's account opened up a conversation about how different members of the family positioned themselves on a semantic polarity of “control” and “chaos” (Ugazio, 2013). This enabled Singh to make an important intervention. Inviting the family to physically take up different positions and then speak from these, she was able to facilitate insight into how another person may be thinking and feeling and in so doing to enable the individuals (and family) to see other, shifting and fluid, positions.

The next session was scheduled as a couple session. Beth arrived, sad but resolute. She said that the last session had made her come to terms with the full impact of Bill's anger on Catherine and Richard and that this had contributed to her acceptance that this “emotional abuse” had to stop. She suggested that Bill temporarily move out of the house, to give the couple and the family a break from this conflict. She was clear, however, that this move was to provide emotional respite and thinking space; she remained committed to continuing couple and family therapy while they were living apart and to finding a long-term solution. Bill remained quiet throughout the session. In this session, Singh returned to Catherine's emotion map to again highlight the impact of his anger on the children. As the Christmas break was coming up, a family session was scheduled for after the holiday period.

**Figure 4:**
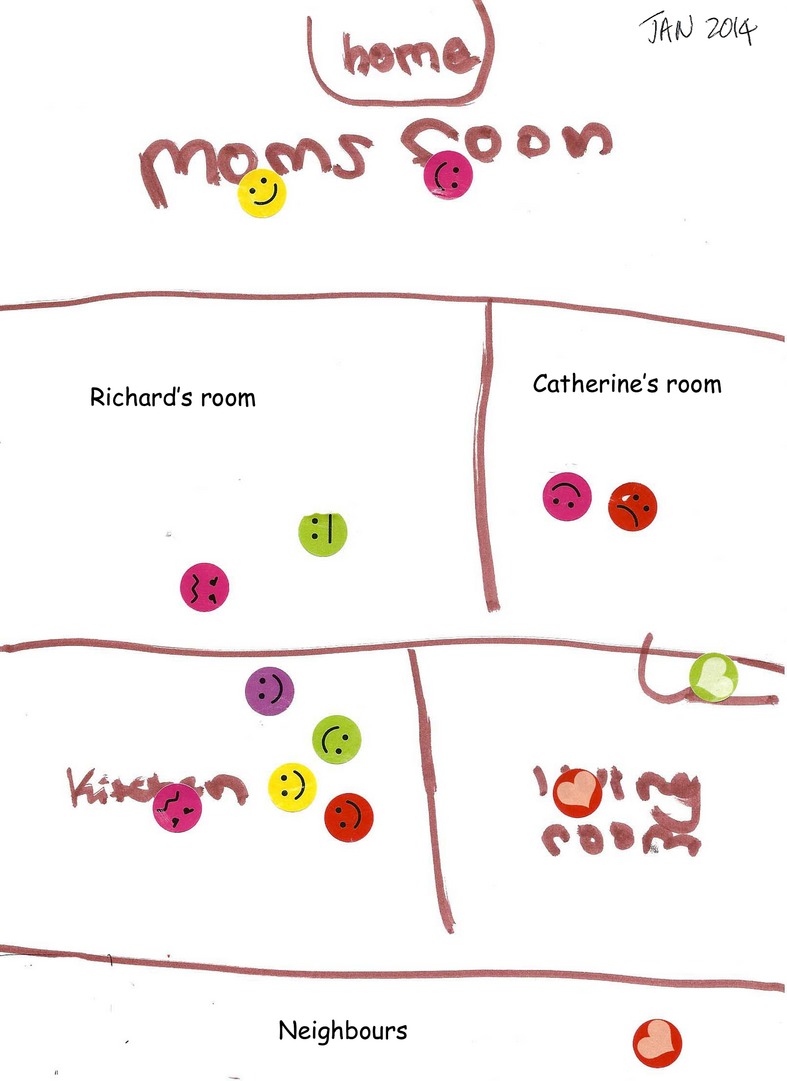
Catherine (Aged 10) Emotion Map 2 Yellow = Dad; pink = Mum; Catherine = red; Richard = green; neighbor = purple

In January, in the family session, everyone reported having enjoyed a harmonious Christmas period, shared with their extended families. During the session, Catherine spontaneously made another emotion map (see Figure 4).

In this second emotion map, the color codes have changed slightly (see key above). In the family session, when she talked about this emotion map, Catherine remarked that Mum was pink, Dad remained yellow, Richard remained green, and she was red. However, the most striking thing about this later emotion map is the change in emotions depicted. Here, Dad is smiling in each of the rooms. Catherine used the heart emoticon in the living room and in the family session she volunteered that “everyone is happy.” The sad and angry faces that are depicted were now attached to particular and transient situations like “Mum is annoyed with Richard because he has stolen my I-pad.” Catherine also talked about being unhappy over an incident that happened in school. There is no depiction of residual bad moods or anger and those that are portrayed are not assigned to the father. Indeed, the family was able to talk and laugh together about the scenarios depicted in the emotion map. For the first time, Catherine talked about their neighbors (who lived below them) and included one of them (purple emoticon) on her emotion map. The wider geographical and emotional environments were thus brought together; the home depicted was a space where everyone was happy.

## Maps and Territories: Reflections on the Case Study

Within the confines of the case study analysis presented here we have focused attention on the usefulness of household emotion maps in systemic clinical practice and how they can be used to answer questions on the particular family dynamic. For example, how may we account for the dramatic change in the Smith family's emotional landscape? What helped the father to change? And, importantly for the argument of this article, did behavior changes have anything to do with the emotion map intervention? There are indications that support this claim. For example, Catherine's second emotion map was an instrumental tool in facilitating family conversation. Being able to subsequently revisit with the parental couple this pictorial device, which clearly displayed the distress of their daughter, enabled them to face up to the scenario, to talk about the changes they wanted to put in place, and to discuss how they understood these changes. Beth stated that positive changes were in part a consequence of her making a firm decision that the couple would need to separate if Bill's anger continued. Bill agreed, although he also attributed being on holiday and things improving at work as significant and contributory factors on his calmer demeanor and its positive impact on the couple and family relationships. Bill and Beth both stated that seeing the stark impact of emotions in the family, as depicted in Catherine's emotion map, had a significant part to play in recognizing and changing their behavior.

In thinking about the changes in the family, we draw on Korzybski's famous adage, “the map is not the territory” (Korzybski, 1933). The tremendous changes which the family made cannot be simply attributed to the therapeutic work intervention of Singh, let alone to the deployment of a tool as simple as the emotion map. The changes probably have far more to do with circumstances outside the consulting room, the families’ resilience, and the strength of the therapeutic relationship. The emotion map merely came at a time when the family could make use of it and was used in conjunction with other representational methods including drawing and the positioning line.

The process of the work with the Smith family puts us in mind of Pittman's (1984) creative use of a range of innovative techniques that have been described as “wet cocker spaniel therapy.” These derive their name from a fascinating case example of a woman who came out of her depressed state during a family therapy session when her cocker spaniel licked her face. Sometimes, when families reach an impasse or snag point, a technique that is out of the ordinary can push the family to change. The technique is thus spontaneous and responds to the therapist's assessment of presenting issues and the family's style. In this case, the emotion map was one among a repertoire of tools, which fitted the family's mode of communication. They enjoyed using it and were able to reflect on the changes in their family relationships. Singh was able to use it to bring it into the therapeutic conversation as a measure, in the first instance, of the impact of the anger and parental conflict on the relationships within the family and in the second instance, on the changes the family had made.

Although it provided a good fit with Catherine's family mostly because of her drawing skills and abilities, Singh has also used the emotion map to good effect in other clinical contexts, with “verbal couples” when “words get in the way.” Visual methods can cut through the cumulative destructive effect of deeply entrenched verbal patterns that instigate and perpetuate conflict. In this context, following the lead of Rober (2009), Singh has used emotion maps as homework tasks, asking couples to take them away and bring them back into the session. She has foregrounded this task by clarifying with each individual what each emoticon means to them. This process of clarification not only ensures that there are shared understandings of both the task and the tools; it also situates emotions at the center of the clinical encounter. This focus on which emotions are selected for different experiences and interactions is most revealing. For example, in another clinical case, the male partner used the “angry” emoticon to describe an incident when he felt unwell. His female partner expressed her surprise at this, and said that she would have used a “sad” emoticon. Talking about this led to an illuminating discussion on how both of them responded differently to difficult situations and how they understood the meaning of anger and sadness in their relationship in quite different ways. It provoked insight for the couple into how they might misunderstand each other and/or articulate similar experiences through different emotional vocabulary. In this way it opened up new channels of communication.

## Discussion: Clinical Implications and Limitations

In this article, we have illustrated how the emotion map method may be used in clinical contexts. Our discussion, however, also raises interesting questions that stretch beyond the methodological. In foregrounding emotions, the technique requires that we consider afresh how emotions are depicted and individually experienced, re-evaluating suppositions of shared understandings (Gabb & Singh, 2014). Can emotions be represented in a way that is universal or is the interpretation of emotions largely dependent on cultural and gendered factors? If we unpick the basis of shared understandings, what is the impact of this on their interactional nature? In this article, we have begun to think through some of these points.

Using emotion maps in Systemic practice inevitably raises underlying dilemmas and questions. Firstly, as Systemic psychotherapists, emotions are understood as relational and performative (Fredman, 2004) rather than located within individuals. Should we, therefore, be asking families and/or couples to complete emotion maps individually or conjointly? Different circumstances and clinical scenarios require perhaps an individually tailored solution that defies any one particular format. Further consideration is also required around the deployment of power and expertise which systemic psychotherapists might have in shaping the emotion maps produced. If we prescribe the emoticons that family members use are we containing the situation and feelings? What if family members feel a mixture of emotions (individually conflicted and/or collectively divergent) at the same time? Multiple stickers can be used to denote conflicting and complex emotions so this issue may be quite readily assuaged. Working from a collaborative transparent stance (Hoffman, 1993), families could be offered the chance to “make” their own emoticons using “blank” stickers upon which they could draw any number of personal emotions. Having a wider range of possible emotions that participants could own would also take into account that an emotional expression may look very different from one culture than to another. A similar strategy could be deployed to open out any spatial containment. Floor maps could extend to the garden, the car, or even surrounding neighborhoods. For example, in her clinical practice, Singh is supporting another couple whose boat is their haven. Arguments and conflict ostensibly take place only when they are in the bricks and mortar of their home. Clinical work with this couple, including emotion maps of their boat as well as their home, is teasing out these located patterns of behavior.

In a different vein, we may also want to pursue close readings of emotion maps in future analysis, situating these in broader cultural contexts. For example, in the first emotion map drawn by Catherine (see Figure 3), a pink heart is embedded in the title, covering the letter “a” in map. The color selected here, pink, is not used to denote any family member, indeed, in this context it seems to function as a cultural signifier. The emotion map title thus serves to imagine the ideal family household—of loving happy pink hearts. It draws on the same idealized representational tools that feature in Catherine's initial drawing, Figure 2: “My family,” which include blue skies, a yellow sunshine, and a smiling contented “happy family” group. Textual analysis of these representational devices could be advanced. Indeed, the subsequent change in colors that feature in the second emotion map—portraying Mum as pink with Catherine selecting red for herself, the color formerly assigned to her mother—invite such interpretation. However, while this change in colors may be ascribed to an unconscious process, this was neither remarked upon by Catherine nor followed up in the session and therefore it remains highly speculative. Such interpretations arguably overstep the argument of this article as they draw on a psychoanalytical mode of interpretation that would require systematic reworking; they stretch the efficacy of emotion maps beyond the scope of their current potential and sit outside the primary foci of this journal. If and when they become more commonly used, they may then present such readings and as such they are highlighted here to flag these future analytical possibilities.

At this point in time, in this article we have aimed to show the uses and application of the emotion map tool, especially in work with couples, children, and families. The novelty and newness of the method inevitably means that it needs to be tested out and adapted if it is to meet its full potential: for example, to explore how it may be used to highlight and work with different contexts, cultures, and corresponding emotions (Gabb & Singh, 2014). Much of the clinical work of Singh with disadvantaged families has been completed through outreach networks in community-based settings. Using emotion maps with these families fits in well with existing good practice which values home visits and working nonverbally in contexts where literacy and verbal communication is an issue.

Emotion maps have also been successfully used by some of Singh's colleagues,^3^ in clinical work with families whose mother tongues are not English and/or with family groups who struggle to express themselves emotionally. Here, they have proven particularly effective in cases where the intensity of feelings appear to overwhelm family members. For example, in a multi-lingual family who had experienced multiple past traumas, using emoticons allowed the children to freely express their thoughts and feelings, which were otherwise hard to verbally articulate. In a session with the teenage children, the idea of using emotion maps was introduced, using emoticons and different colored marker pens for each feeling experienced. The young people were quick to grasp the concept and indeed one of them drew his own emoticons to illustrate the particular feelings that had been individually identified. Placing emoticons in the living spaces which they inhabited and attributing different emoticons to different family members enabled them to think about the context for these emoticons, including times of day, who was present, who had similar emotions, and who did not. The discussion of emotions opened up the therapy which was in danger of shutting down due to difficulties experienced in verbal emotional expression. In talking about everyday emotions, a route into the feelings that are more specific to the past traumas was also opened up. Using emoticons in a more curious way, the discussion of emotions in general has provided a useful way into the feelings that are more specific to the past traumas.

There are possible practical considerations that may delimit widespread use of emotion maps. At present, emoticons can be individually designed and ordered through print services but they are not readily available to the general population. The clinical work of Singh illustrates that the polished artifacts developed in research are not necessary; indeed, the immediacy of the method may be part of its appeal. Singh and colleagues do nevertheless have access to sheets of stickers and these have facilitated the process. (Emoticon sticker sheets can also be obtained by contacting author Jacqui Gabb). A multi-platform emotion map App is under development. This will be freely available and thus increase its potential usage and appeal—especially among younger populations. Given the number of couples who now live apart together (Duncan & Phillips, 2010) or who lead incredibly busy work lives which take them away from home for sustained periods of time, an emotion map App will make a valuable adjunct to more conventional methods from family therapy practice. In these scenarios, digitally mediated communication in the form of Skype, emails, and texts are a crucial part of couple communication (Gabb, Klett-Davies, Fink, & Thomae, 2013, p. 51) and family processes more generally. Indeed, Singh was immediately captivated by emotion maps because they resonated with her—both personally and professionally. Their professional appeal in work with families and emotions has been discussed above; but it was more than this, too. It was the format of this method that also set it apart. At a personal level, a lot of her family communication took place via texts and emails. When communicating good news, for example, such as a successful exam or job interview, a smiling face would be added; whereas bad news was accompanied by a sad face. Emotion maps thus seem to neatly sit at the interface between technology, family life, and communication and have significant capacity in clinical assessment and practice.

## Footnotes

1 *Behind Closed Doors. Researching Intimacy and Sexuality in Families* (RES-000-22-0854). *Enduring Love? Couple Relationships in the 21st Century* (RES-062-23-3056). Both studies were funded by the Economic and Social Research Council and completed in the UK. *Behind Closed Doors* was a methodological pilot project designed to interrogate the efficacy of multiple qualitative methods in studying personal relationships and family lives (*n* = 11). *Enduring Love?* examined how couples sustain long-term relationships (http://www.enduringlove.co.uk). The project included a large-scale survey (*n* = 5494) and qualitative multiple methods research (*n* = 50). The research design of the *Enduring Love?* project was developed in consultation with key UK relationship support organizations.

2 All names and identifying details have been changed to protect confidentiality. The family has given their informed consent for Catherine's emotion maps to be used in this case example.

3 The example included here comes from the clinical practice of Systemic Psychotherapist Doreen Robinson.
